# Predicting survival in metastatic non‐small cell lung cancer patients with poor ECOG‐PS: A single‐arm prospective study

**DOI:** 10.1002/cam4.5254

**Published:** 2022-09-26

**Authors:** Mateus Trinconi Cunha, Ana Paula de Souza Borges, Vinicius Carvalho Jardim, André Fujita, Gilberto de Castro

**Affiliations:** ^1^ Serviço de Oncologia Clínica, Instituto do Câncer do Estado de São Paulo, Hospital das Clínicas HCFMUSP, Faculdade de Medicina Universidade de São Paulo São Paulo Brazil; ^2^ Faculdade de Medicina FMUSP Universidade de São Paulo São Paulo Brazil; ^3^ Departamento de Ciência da Computação, Instituto de Matemática e Estatística Universidade de São Paulo São Paulo Brazil

**Keywords:** machine learning, network analysis, non‐small cell lung cancer, poor performance status, prognostic markers

## Abstract

**Background:**

Patients with advanced non‐small cell lung cancer (NSCLC) are a heterogeneous population with short lifespan. We aimed to develop methods to better differentiate patients whose survival was >90 days.

**Methods:**

We evaluated 83 characteristics of 106 treatment‐naïve, stage IV NSCLC patients with Eastern Cooperative Oncology Group Performance Status (ECOG‐PS) >1. Automated machine learning was used to select a model and optimize hyperparameters. 100‐fold bootstrapping was performed for dimensionality reduction for a second (“lite”) model. Performance was measured by C‐statistic and accuracy metrics in an out‐of‐sample validation cohort. The “lite” model was validated on a second independent, prospective cohort (*N* = 42). Network analysis (NA) was performed to evaluate the differences in centrality and connectivity of features.

**Results:**

The selected method was ExtraTrees Classifier, with C‐statistic of 0.82 (*p* < 0.01) and accuracy of 0.81 (*p* = 0.01). The “lite” model had 16 variables and obtained C‐statistic of 0.84 (*p* < 0.01) and accuracy of 0.75 (*p* = 0.039) in the first cohort, and C‐statistic of 0.706 (*p* < 0.01) and accuracy of 0.714 (*p* < 0.01) in the second cohort. The networks of patients with lower survival were more interconnected. Features related to cachexia, inflammation, and quality of life had statistically different prestige scores in NA.

**Conclusions:**

Machine learning can assist in the prognostic evaluation of advanced NSCLC. The model generated with a reduced number of features showed high accessibility and reasonable metrics. Features related to quality of life, cachexia, and performance status had increased correlation and importance scores, suggesting that they play a role at later disease stages, in line with the biological rationale already described.

## Graphical abstract

A machine learning (ML) model was developed to predict whether patients with advanced NSCLC and ECOG‐PS >1 would have an overall survival (OS) >90 d. Thereafter, a lite model with 16 easily accessible features was created, and its prospective validation yielded a C‐statistic of 0.706 (*p* < 0.01) and accuracy of 0.714 (*p* < 0.01). The features used in the ML model and network analysis of significant relations between features suggest that inflammation, QoL and cachexia play a major role in determining OS in these patients.

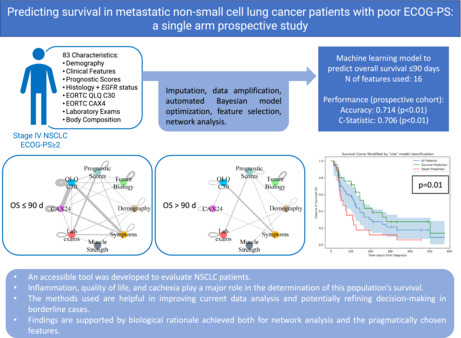

## 1 INTRODUCTION

Recent data suggest a wide variation of survival among patients with metastatic non‐small cell lung cancer (NSCLC), the leading cause of cancer‐related deaths worldwide, largely attributable to the clinical and molecular heterogeneity of this population.^1^

Most prospective studies exclude patients with poor PS (ECOG‐PS > 1), especially in the late‐stage setting, when it occurs in 48% of NSCLC patient self‐reports.^2^ The risk of aggressive treatment causing harm in poor ECOG‐PS NSCLC patients is not negligible.^3^, ^4^ Besides, the early introduction of palliative care was also shown to improve clinical outcomes and reduce financial burden.^5^, ^6^

Predicting survival is crucial to determining best resource allocation, treatment objectives, and advanced healthcare directives.^7^ Clinician‐made survival estimation alone is reported to be overly optimistic.^4^, ^8^, ^9^ Several prognostic models have been developed to estimate the survival of terminally ill cancer patients. The currently established models generally present either a lack of clinical application, no or minimal external validity,^10^ not being developed with a specific primary cancer, or not considering nuances of cancer stages.^11^, ^12^, ^13^ In addition, most models rely solely on assessing one clinical dimension (e.g. symptoms, functionality, or disease burden).

More recently, machine learning (ML) models and nomograms have become more popular tools for the task of outcome predictions. These methods allow for assessing complex associations between variables, surpassing the performance of previous models crafted through analog means, or even providing treatment recommendations.^14^, ^15^, ^16^, ^17^ However, there is limited work on evidence supporting accurate prediction for NSCLC patients with poor PS.

Thus, more extensive research is required to develop a model capable of assisting healthcare providers in better caring for this vulnerable population of poor PS advanced NSCLC patients and allocating resources to reduce the disease's burden to patients, families, and taxpayers.

The aim of this study is to extensively assess demographic, clinical, and laboratory features of metastatic NSCLC patients with poor PS, analyze the interaction between them, and evaluate the performance of a patient classification model to predict overall survival (OS) ≤90 days using automated ML optimization strategies.

## 2 MATERIALS AND METHODS

### 2.1 Patients

Patients enrolled in our institution from 19 November 2017, to 1 February 2021 (cut‐off date) were included in this prospective, single‐center study. Eligible patients were adults 18 years and older, with histologically confirmed, treatment‐naïve, advanced NSCLC (American Joint Committee on Cancer [AJCC] 7th edition IVa or IVb stages), with ECOG‐PS 2, 3, or 4.

The patients were collected in two different moments: the first (Cohort 1 [C1]) was collected as a discovery cohort for model development. Alternatively, the second (Cohort 2 [C2]) was collected posteriorly to the completion of the development of our ML model, as a second out‐of‐sample dataset for independent validation.

### 2.2 Patient features

We collected 83 baseline features about demography, histology, metastatic sites, *EGFR* mutational status; nutritional status (body mass index [BMI] and weight loss in the last 6 months), and body composition (mean hand grip strength,^18^ dominant mid‐arm muscle circumference and measurement of the psoas as well as abdominal intermuscular adipose tissue, subcutaneous adipose tissue, and visceral adipose tissue^19^, ^20^); medical history (comorbidities, Charlson Comorbidity Index^21^, ^22^), smoking status, PS (ECOG‐PS^23^ and Karnofsky scales^24^); symptoms, Edmonton Symptom Assessment System (ESAS)^25^ and the Modified Medical Research Council dyspnea classification (mMRC); strong opioid use and oxygen supplementation; European Organization for Research and Treatment of Cancer Quality of Life Questionnaire (EORTC‐QLQ) Core 30^26^ and CAX24,^27^ palliative scores (Palliative Performance Index [PPI]^13^ and Palliative Prognostic Score [PaP]^12^), and laboratory values (blood cell counts, renal function, C‐reactive protein, and albumin). We disconsidered C‐reactive protein and albumin because of a high percentage of missing values.

### 2.3 Survival analysis

The median OS (mOS) was determined by performing a Kaplan‐Meier OS analysis of all 106 patients.

### 2.4 Data pre‐processing

We scaled the data of C1 by minimal and maximum feature values (MinMax scaling), ranging from 0 to 1. We imputed the missing data using the K‐nearest‐neighbors (KNN) method. The KNN‐based imputation is a classic method that selects K patients nearest to the patient with the missing data. The distance was determined in Manhattan distance, measured by each patient's position on a multidimensional graph, in which each dimension is a feature. We then used a weighted average of values to estimate the missing value.^29^ We weighted the K patients based on the similarity of their features to those of the patient of interest. After imputation, to avoid data leakage, scaling was undone. We labeled data as the OS status via one‐hot encoding, being patients with OS >90 days “0” and the remaining, “1.” We split a validation set (Cohort 1.5 [C1.5]) by randomly selecting 16 (15.09%) patients.

### 2.5 Outlier removal

After separating C1.5, we removed the outliers in the 90 patients remaining in C1. We did this by grouping patients with the same label (i.e. 1 or 0). Then, we clustered them using KNN (after another MinMax scaling). Finally, we used Density‐Based Spatial Clustering of Applications with Noise (DBSCAN)^29^ to remove those who did not belong to any cluster. This process resulted in the removal of six patients, three from each label group, and leaving 84 patients in C1.

### 2.6 Data balancing

To reduce possible imbalances within C1, the 84 data points underwent Synthetic Minority Over‐Sampling Technique (SMOTE). This approach amplifies data synthetically, especially in small data sets, generating new, artificial patients similar to those of the minority group.^31^ This method yielded 12 new data points, resulting in 96 patients for training and testing. After SMOTE, MinMax scaling was undone, returning all features to their standard units.

### 2.7 Model selection

We used Hyperopt (documentation available at https://hyperopt.github.io/) to select and optimize the ML classification model, preprocessing methods (scaling and/or normalization), and their hyperparameters.^32^, ^33^ This is a Bayesian optimization open‐source python library that comprises iterative probability model updating to maximize model performance, in this case, measured as C‐statistic. The classifier models tested were as follows: C‐Support Vector Classification, K‐nearest Neighbors, Adaptive Boosting, Gradient Boosting, Random Forest, Extremely Randomized Trees, Decision Tree, Stochastic Gradient Descent, Extreme Gradient Boosting, Naïve Bayes, Passive Aggressive, Linear Discriminant Analysis, Quadratic Discriminant Analysis, One versus Rest, and One versus One. Preprocessing methods tested were as follows: Principal Component Analysis, One Hot Encoder, Standard Scaler, Min Max Scaler, Normalizer, and Restricted Boltzmann Machine.

### 2.8 Training and test sets

We split C1 randomly 100 times into different training and test dataset iterations to estimate the model's performance and eliminate sample selection bias. The test sets, used to assess the model's performance immediately after training, contained a random population consisting of 20% of the C1 (*n* = 19). We assigned the remaining 80% (*n* = 77) to the training set. We used the training set to tune the parameters of the model.

### 2.9 Model performance analysis

The model defined and optimized by HyperOpt was trained with each of the 100 different training sets and tested with the respective test sets. We used the mean area under the receiver operating characteristic curve (ROC) (C‐statistic) and accuracy to measure model performance. We considered statistically significant the ML metrics with *p*‐values <0.05, as calculated through the Monte Carlo Method.

### 2.10 Significant feature selection for “lite” model building

**FIGURE 1:**
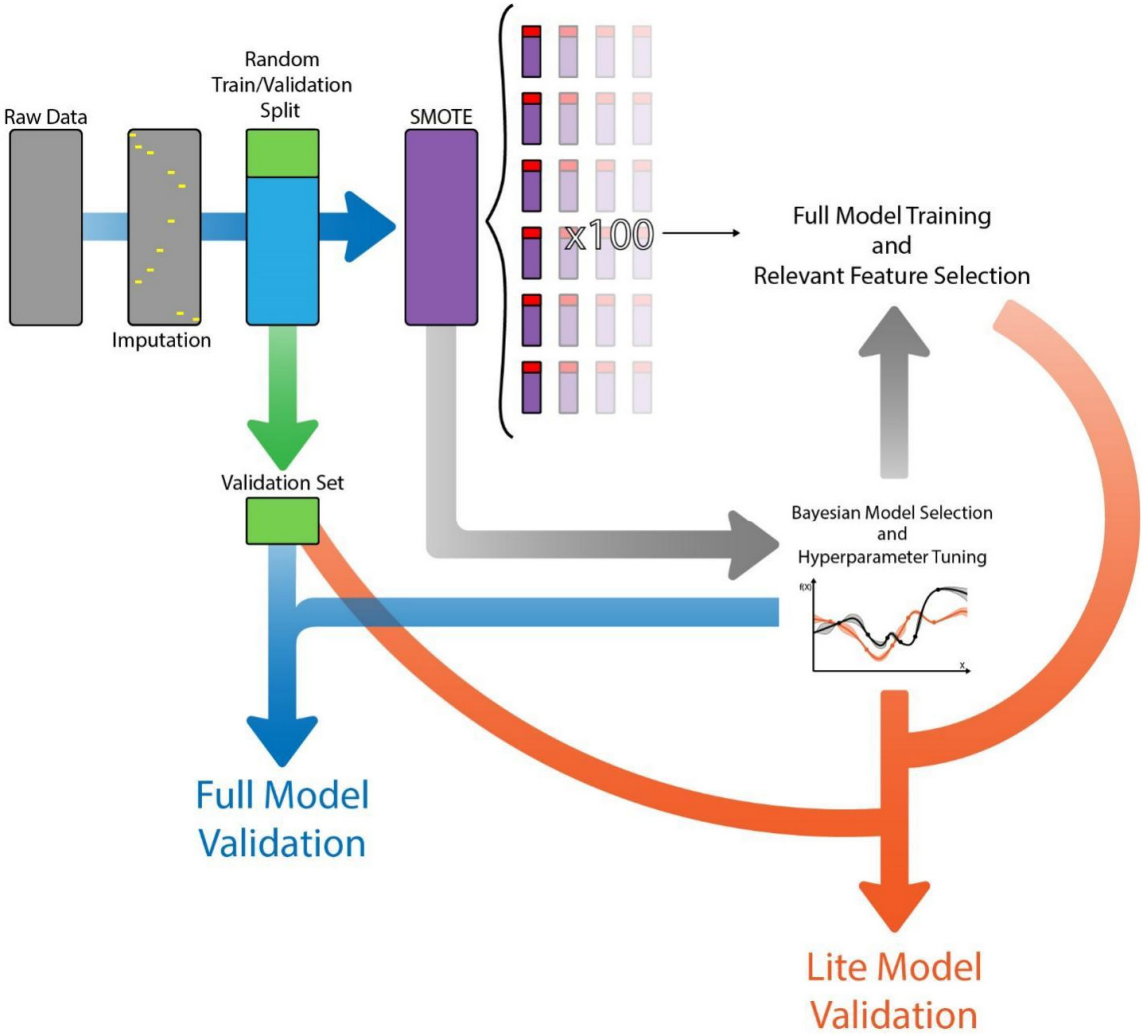
Diagram of the data pipeline from raw data to machine learning model validation metrics in the C1 data set. The raw data were submitted to imputation, followed by splitting of 16 patients into the validation set (C1.5). We performed Synthetic Minority Over‐Sampling Technique (SMOTE) on the remainder of the dataset for data balancing and then made a 100× bootstrap, generating 100 different combinations of training and test datasets. The original dataset with synthetic data points was also used to train the Bayesian model selection and hyperparameter tuning library (HyperOpt). The selected and tuned model was trained and tested in each bootstrapped dataset with all features (full model), with the selection of the most relevant features. Validation on C1.5 was also performed with the full model. The same classifier and hyperparameters were trained and tested in filtered versions of the bootstrapped datasets. These filtered versions contained only the features selected during the training of the full model. The model with a reduced number of features (“lite” model) was then tested on a filtered C1.5

A smaller number of features (ideally the least possible) are preferred to increase the model's ease of use and generalization to predict previously unseen data. For this purpose, each iteration of the selected model voted for the most significant features. We used those selected by over 60% of the iterations for further analysis. A collinearity assessment was made by calculating variable inflation factors and Spearman's correlation coefficient matrix, excluding the least voted features of highly correlated groups. The remaining variables composed the final selection for the “lite” model. Its performance was measured by the mean area under the ROC curve (C‐statistic) and accuracy in the 100‐fold bootstrap of C1 and validation with C1.5. We performed a Cox analysis to verify the relationship between each variable selected for the “lite” model with patients' survival. Figure 1 presents a diagram of the data pipeline from raw data to results in C1 and C1.5.

After validation with C1.5, the “lite” model was trained with a dataset comprising the modified C1 (after imputation, outlier removal, and SMOTE) and C1.5 (the pure, unadulterated validation data removed from C1 prior to modifications). This version of the “lite” model was used to predict the survival of the patients prospectively collected in C2. Model performance was measured by the mean area under the ROC curve (C‐statistic) and accuracy.

### 2.11 Network analysis

We used the raw C1 dataset to avoid false discoveries caused by deleting any feature with more than 5% missing values and consequent data imputation. Each node represents a feature. The correlation between all feature pairs was calculated by Spearman's correlation coefficient. We corrected the *p*‐values for multiple tests using the false discovery rate (FDR) Benjamini‐Hochberg method.^33^ After correlation selection, we plotted network graphs using force‐directed (Fruchterman‐Reingold) and circular layouts. The assessment of node centrality in the constructed network was made by eigenvector centrality analysis. A higher prestige score indicates that one node (or feature) correlates to other influential nodes and vice versa.^34^ Several fields of information science use this method, such as modern search engines.^35^

We built one network for each group of patients (OS ≤90 days and >90 days). We used BioNetStat to verify if there were statistical differences between the groups' networks. To this end, we statistically compared their structures (spectral entropy) and the prestige score of features (eigenvector centrality).^36^ We also performed an edge statistical comparison between the networks of OS groups. After determining significant correlations in each network (corrected *p*‐value <0.05), we compared these correlations in the two survival groups using bootstrap analysis. By correcting the *p*‐value of the bootstrap analysis by FDR, we got the valid correlations that statistically change between the two groups.

To reduce the number of variables, we sorted them into groups: QLQ C30, CAX 24, Prognostic Scores, Laboratory Exams, Muscle Strength, Symptoms (ESAS), Demographic Characteristics, and Tumor Status. Each group is a node. One group is connected to another if the features from both groups are connected. The weight of edges between groups is the sum of edges' weight that connects groups' features. We show a loop edge connecting the group node to itself when the connected variables are in the same group.

### 2.12 Ethical considerations

This prospective study “Factors Determining the Prognosis in NSCLC Stage IV (LUPROG)” (NCT04306094) was approved by the local ethics committee. Informed consent was obtained from all participants. The present study follows the Guidelines for Developing and Reporting ML Predictive Models in Biomedical Research for the transparent report of an ML architecture for individual prognosis estimation.^38^ The data generated and analyzed during this study are available from the corresponding author upon reasonable request.

## 3 RESULTS

### 3.1 Patients

One hundred and six patients were included in C1. The median (interquartile range) age was 66 years old (59–71), 45% were male, 65% were Caucasian, and 84% were smokers (41 pack‐years [20–60]). We detected *EGFR* activating mutations in seven (7/62 [11%] tested) patients. However, 44 patients did not have their *EGFR* mutational status assessed. Median BMI was 22.09 kg/m^2^ (19.46–24.44), with a median 6‐month weight loss of 10.5 kg (7–15). Fifty‐eight (54.7%) patients had ECOG‐PS 2, 36 (33.9%) ECOG‐PS 3, and 12 (11.3%) ECOG‐PS 4. Fifty patients (47.1%) needed supplemental oxygen, and 24 (22.6%) used potent opioids. The full C1 mOS was 65 days (29–180), and 60 patients (56%) lived at least 90 days.

Forty‐two patients were included in C2. The median (interquartile range) age was 68 years old (64.5–69.5), 56% were male, 73% were Caucasian, and 69% were smokers (42 pack‐years [38–74]). We detected *EGFR* activating mutations in five (5/41 [12%] tested) patients. However, 19 patients did not have their *EGFR* mutational status assessed. Median BMI was 21.34 kg/m^2^ (19.28–25.27), with a median 6‐month weight loss of 10.5 kg (7–15). Twenty‐five (61.9%) patients had ECOG‐PS 2, 13 (30.9%) ECOG‐PS 3, and three (7.1%) ECOG‐PS 4. Five (11.9%) needed supplemental oxygen, and 19 (45.2%) used potent opioids. The full cohort mOS was 119 days (confidence interval [CI] 95% 67–168), and 25 patients (59.5%) lived at least 90 days.

Table 1 contains a more extensive description of the two cohorts and a statistic comparison between their features.

**TABLE 1:** Characteristics of the patients included in the study and *p*‐values for the comparison between Cohorts 1 and 2. EGFR status was only assessed with adenocarcinoma histology

Characteristic	Cohort 1			Cohort 2			*p*‐value
	*N* (%) or median (IQR)						
*N*	Total (*N* = 106)	OS >90 days (*N* = 47)	OS ≤90 days (*N* = 59)	Total (*N* = 42)	OS >90 days (*N* = 25)	OS ≤90 days (*N* = 17)	
OS (days)	64 (29–180)	199 (142.5–295)	30 (16–52)	119 (67–168)	165 (103–280)	61 (38–115)	0.429
Demography							
Age (years)	66 (59–71)	67 (59–71)	66 (59–71)	68 (65–70)	65 (60–70.6)	69 (64–75)	0.7133
Active tobacco use	90 (84%)	39 (82.9%)	51 (86.4%)	29 (69%)	17 (68%)	13 (76.4%)	0.06
Tobacco burden (pack‐years)	41 (20–60)	40 (12.5–60)	45 (25–60)	40 (18–58)	30 (7.5–45)	56 (38–75)	0.403
White	68 (64.2%)	27 (57.4%)	41 (69.5%)	31 (73.8%)	18 (72%)	13 (76.4%)	1
Black	11 (10.3%)	5 (10.6%)	6 (10.1%)	4 (9.5%)	2 (8%)	2 (11.7%)	
Sex							0.2069
Female	58 (55%)	17 (36.2%)	30 (50.8%)	18 (42.9%)	14 (56%)	4 (23.5%)	
Male	48 (45%)	30 (63.8%)	29 (49.2%)	24 (57.1%)	11 (44%)	13 (76.4%)	
Pathology/EGFR status							
Adenocarcinoma	72 (68.0%)	35 (74.5%)	37 (62.7%)	23 (54.8%)	16 (64%)	7 (41.2%)	0.1084
Squamous Cell Carcinoma	20 (18.8%)	9 (19.1%)	11 (18.6%)	15 (35.7%)	8 (32%)	7 (41.2%)	
Undifferentiated	14 (13.2%)	3 (6.4%)	11 (18.6%)	4 (9.5%)	1 (4%)	3 (17.6%)	
wt*EGFR*	47 (44.3%)	24 (51.1%)	23 (39.0%)	8 (19%)	6 (24%)	2 (11.8%)	0.042
mut*EGFR*	7 (6.7%)	3 (6.3%)	4 (6.8%)	5 (11.9%)	4 (16%)	1 (5.9%)	
*EGFR* inconclusive	8 (7.5%)	5 (10.6%)	3 (5.1%)	3 (7.1%)	2 (8%)	1 (5.9%)	
*EGFR* unknown	10 (9.4%)	3 (0.64%)	7 (11.9%)	7 (16.7%)	4 (4%)	3 (17.6%)	
ECOG‐PS							0.6612
2	58 (54.7%)	33 (70.2%)	25 (42.4%)	26 (61.9%)	17 (68%)	9 (52.9%)	
3	36 (33.9%)	11 (23.4%)	25 (42.4%)	13 (31%)	8 (32%)	5 (29.4%)	
4	12 (11.3%)	3 (6.4%)	9 (15.2%)	3 (7.1%)	0	3 (17.7%)	
Metastasis site							0.3043
Central Nervous System	25 (23.6%)	11 (23.4%)	15 (23.7%)	10 (23.8%)	5 (20%)	5 (29.4%)	
Bone	43 (40.6%)	21 (44.7%)	22 (37.3%)	18 (42.9%)	10 (40%)	8 (47.0%)	
Contralateral lung	34 (32.1%)	18 (38.3%)	16 (27.1%)	11 (26.2%)	7 (28%)	4 (23.5%)	
Pleura	26 (34.0%)	17 (36.2%)	19 (32.2%)	13 (31%)	8 (32%)	5 (29.4%)	
Carcinomatous lymphangitis	5 (4.7%)	2 (4.3%)	3 (5.1%)	5 (11.9%)	3 (12%)	2 (11.7%)	
Liver	11 (10.4%)	1 (2.1%)	10 (16.9%)	3 (7.1%)	1 (4%)	2 (11.7%)	
Adrenal	27 (25.5%)	10 (21.3%)	17 (28.8%)	5 (11.9%)	3 (12%)	2 (11.7%)	
Clinical features							
Charlson score	8.5 (8–10)	9 (8–9.5)	8 (8–10)	9 (8–9)	9 (8–9)	9 (8–10)	
Modified Medical Research Council	3 (1–4)	2 (1–3)	3 (2.5–4)	0.5 (0–4)	0 (0–3)	3 (0–4)	
Strong opioid use	50 (47.2%)	13 (27.7%)	37 (62.7%)	19 (45.2%)	10 (40%)	9 (52.9%)	0.8569
Supplemental oxygen need	24 (22.6%)	2 (4.3%)	22 (37.2%)	5 (11.9%)	1 (4%)	4 (23.5%)	0.2476

### 3.2 Model selection

The model selected and adjusted by the automated ML library was Extremely Randomized Trees Classifier (ExtraTreesClassifier), and no preprocessing (scaling/normalization) was necessary. Figure S1 shows the performance of the 20 best models assessed.

### 3.3 “Lite” model feature selection

The initial model was built with 83 features (listed in the methods sections), and only 16 were included after feature selection. The ones judged the most important after bootstrapping were as follows: need for supplementary oxygen, strong opioid use, EORTC‐QLQ C30 physical functioning, and role functioning. Hematocrit, ECOG‐PS, wild‐type *EGFR* status, EORTC CAX24 “dry mouth,” and PaP score were selected as significant by at least 93% of iterations (Table 2).

**TABLE 2:** Significant features and number of times each of them were selected as significant by the iterations. Maximum number of votes = 100. Hemoglobin and leukocyte count were later discarded for high collinearity with other variables

Feature	Votes
Supplemental oxygen need	100
Strong opioid use	100
QLQ‐C30 physical functioning	100
QLQ‐C30 role functioning	100
Hematocrit	99
ECOG‐PS	98
wtEGFR	96
CAX24 dry mouth	94
PaP score	93
Dominant mid‐arm circumference	87
QLQ‐C30 appetite loss	86
CAX24 loss of control	85
CAX24 food aversion	82
Contralateral lung metastasis	80
Neutrophil count	78
mMRC	76
Hemoglobin	70
Leucocyte count	64

### 3.4 All features and “lite” model performances

The model trained with all 83 variables achieved a mean C‐statistic of 0.818 (median 0.830, 95% CI 0.800 to 0.836), and mean accuracy of 0.725 (median 0.750, 95% CI 0.705 to 0.745) in C1. Validation scores in C1.5 were C‐statistic of 0.817 (*p* = 0.0021) and accuracy of 0.813 (*p* = 0.0107).

The “lite” model (with the 16 selected features) achieved a mean C‐statistic of 0.851 (median 0.862, 95% CI 0.835 to 0.867) and mean accuracy of 0.760 (median 0.750, 95% CI 0.742 to 0.778) in C1. Validation scores in C1.5 were C‐statistic of 0.843 (*p* = 0.0021) and accuracy of 0.750 (*p* = 0.039). Validation scores in C2 were C‐statistic of 0.706 (*p* = 0.0034) and accuracy of 0.714 (*p* = 0.0069).

**FIGURE 2:**
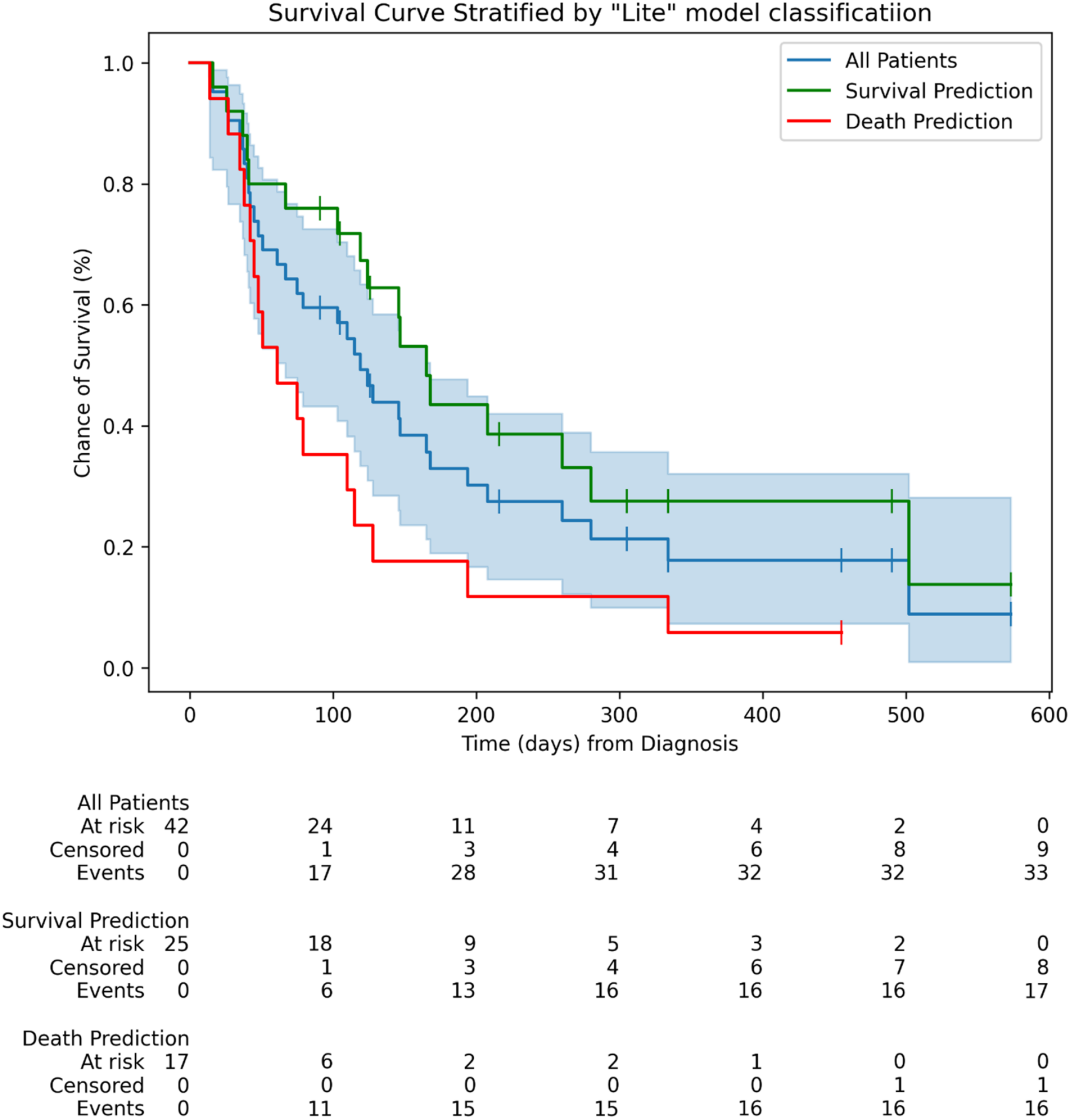
Kaplan‐Meier survival estimates for all C2 patients (blue) with its 95% confidence interval (light blue area) and survival lines of C2 patients classified by the “lite” model as predicted OS >90 days (green) and ≤90 days (red). Combined with the log‐rank test performed, this image illustrates how machine learning‐assisted assessment can be an important factor for the prediction of patient survival

Patients from C2 classified as predicted OS >90 days had a mOS (CI 95%) of 165 days (103–280), while that of those classified as predicted OS ≤90 was 61 days (38–115). Log‐rank analysis shows a significant difference between estimated survival of different groups (*p* = 0.01). Figure 2 shows a Kaplan‐Meier plot of the survival of the patients in C2.

In the Cox analysis with 16 features selected for the “lite” model, six were related to patients' survival (*p*‐value <0.05): CAX24 features (dry mouth, loss of control, and food aversion), strong opioid use, ECOG‐PS, and PaP score (Table 3).

**TABLE 3:** Hazard ratios and CI 95% for the features selected by the “lite” model. Variables marked with a “‐yes” took their absence as reference

Feature	Hazard ratio	*p*‐Value
ECOG‐PS (reference: ECOG‐PS 2)		
ECOG‐PS—3	0.8 (0.44–1.46)	0.471
ECOG‐PS—4	2.98 (1.08–8.22)	0.035
EGFR status (reference: wild type)		
EGFR status—mutated	0.74 (0.29–1.89)	0.525
EGFR status—inconclusive	1.11 (0.62–1.98)	0.722
PaP Score	1.66 (1.08–2.55)	0.02
QLQ‐C30 physical functioning	1.04 (0.73–1.47)	0.842
QLQ‐C30 role functioning	0.77 (0.55–1.09)	0.138
QLQ‐C30 appetite loss	0.91 (0.61–1.36)	0.643
Supplementary oxygen need—yes	1.73 (0.83–3.62)	0.143
Strong opioid use—yes	2.4 (1.39–4.12)	0.002
CAX24 dry mouth	0.72 (0.53–0.97)	0.031
CAX24 loss of control	0.68 (0.49–0.95)	0.024
CAX24 food aversion	1.51 (1.05–2.16)	0.026
Hematocrit	2.01 (0.51–7.89)	0.317
Hemoglobin	0.32 (0.08–1.28)	0.108
Neutrophil count	1.24 (0.43–3.55)	0.69
mMRC	0.8 (0.58–1.10)	0.166
Leucocyte count	0.96 (0.35–2.63)	0.933
Dominant mid‐arm circumference	1.05 (0.78–1.42)	0.74
Contralateral lung metastasis—yes	1.32 (0.74–2.36)	0.355

### 3.5 Network analysis

Features with more than 5% missing values were removed from the analysis, and after that, patients with any missing data were excluded (Figure S2). The remaining dataset consisted of 73 features and 95 patients. For patients who survived >90 days, 28 correlations between 34 variables were statistically significant. We formed a sparse network with 11 different small networks, each with two to five components. For the cohort of patients who survived ≤90 days, 99 correlations between 54 variables were statistically significant. The correlation among features in this group of patients formed one large network with 51 nodes. The 10 most “important” variables considered by the prestige score (eigenvector centrality) were QLQ C30 (fatigue and physical functioning) and CAX24 (forcing oneself to eat, loss of control, and physical decline). The remainder were of diverse natures: KPS, PPI, hematocrit, hemoglobin, and strong opioid use.

#### 3.5.1 Network comparison

There were statistical differences between networks from OS ≤90 days patients and OS >90 days patients (network centrality *p*‐value = 0.21, network structure *p*‐value = 0.01) in the eigenvector centralities and the structural features of the networks (spectral distribution) (Table S1).

When comparing the weight of edges between two networks, nine edges (correlation lines between nodes) remain statistically significant (Figure S3). The QLQ C30 social functioning, CAX24 (eating difficulties, eating and weight‐loss worry, and forcing oneself to eat), blood analysis (hematocrit, hemoglobin, leucocyte, and neutrophil counts), prognostic scores, weight loss, and strong opioid use show changes in correlations.

#### 3.5.2 Feature groups networks

**FIGURE 3:**
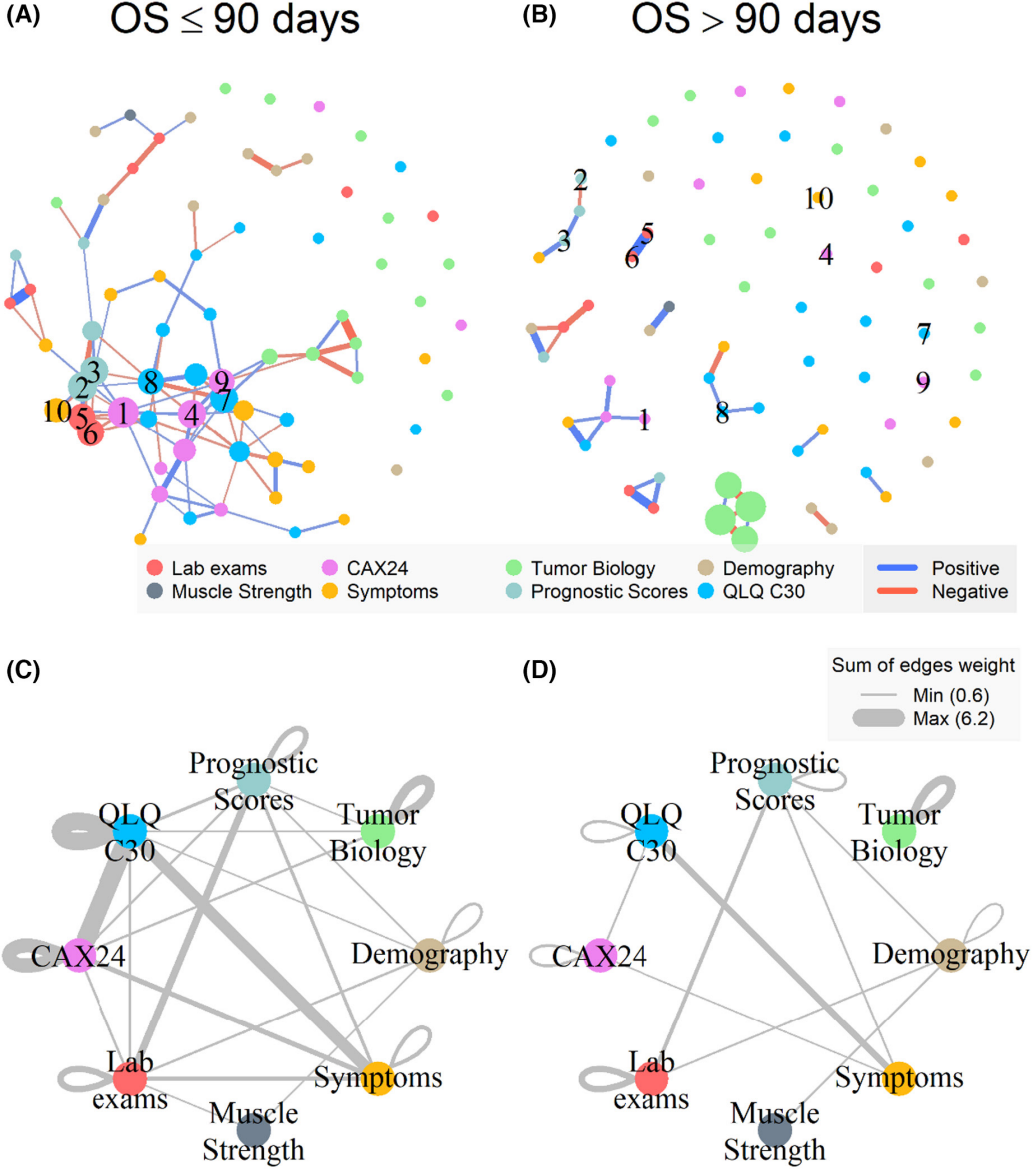
Network analysis representation for patients with OS ≤90 days (A, C) and >90 days (B, D). All features are represented in panels A and B without names for easier visualization. The network with feature names is in Figure S4. Edge color and width represent the sign and magnitude of correlations, respectively. Node radius represents prestige score. The numbers in panels A and B are the 10 higher prestige scores of features in OS ≤90 days network patients: 1: CAX24 Forcing Self to Eat; 2: KPS; 3: PPI; 4: CAX24 Loss of Control; 5: Hematocrit; 6: Hemoglobin; 7: QLQ‐C30 Fatigue; 8: QLQ‐C30 Physical Functioning; 9: CAX24 Physical Decline; 10: Strong Opioid. In panels C and D, the nodes are features grouped by similarity. Edge width represents the sum of the absolute values of correlations between features

The grouping analysis of networks shows that the OS ≤90 days (Figure 3A,D) network has more connections in comparison to >90 days (Figure 3B,D). The QLQ C30 and CAX24 groups in the OS ≤90 days network share a high‐weighted edge. On the other hand, QLQ C30 and ESAS Symptoms share a high‐weighted edge in the OS >90 days network.

## 4 DISCUSSION

In this study, we gathered multidimensional data from 106 patients with metastatic NSCLC and ECOG‐PS >1, and created a ML model with a C‐statistic of 0.843 and an accuracy of 0.750 in the discovery cohort and respectively 0.706 and 0.714 for an out‐of‐sample, completely prospective cohort, while using only 16 easily assessable patient characteristics, mainly related to quality of life, symptoms, and cachexia. The results indicate that we can create a model with acceptable performance with modern data imputation and amplification methods, as well as hyperparameter optimization, compensating for a limited number of training data points.

Here, we present a prospective design, seldom seen in ML studies. In addition, this is a comprehensive assessment of several biological, clinical, and psychosocial aspects of patients. It also uses numerous modern data analysis tools to study this population, producing valuable results in an out‐of‐sample dataset. We can explain the discrepancy between testing and validation metrics in the complete model by sample selection bias: due to the small size of the dataset, larger variations are prone to occur, and the sample used might have been one in which, coincidentally, the selected model would perform better than 95% CI. After feature selection, this phenomenon seemed to be corrected, returning the accuracy within a range of the 95% CI of the training set, remaining within reasonable metrics. This might be justified by the removal of statistical noise, which possibly contributed to reducing selection bias. The reduction in dimensionality also contributed to the accessibility of the model. Finally, a drop in C‐statistic and accuracy occurred when testing in C2, as is to be expected in external validation groups, especially considering the size of the prospective cohort, as large as 39.6% of C1.

Another noteworthy point is the possibility of pragmatic variable selection for prognostic evaluation. This often results in optimal ML performance with a more compact set of features.^39^ The model we developed shows that clinically deteriorated, cachectic, with inferior quality of life scores, wild‐type *EGFR*, and with poor prognostic predictors tend to be classified as short OS (≤90 days), showing biological plausibility, related to cancer‐induced systemic inflammation.^40^ Of note, when analyzing the “lite” model features in a multivariate model, only 6 of the 16 selected variables have a statistically significant hazard ratio. However, this analysis only shows differences in the whole cohort. Decision‐tree‐based methods such as ExtraTreesClassifier change population composition after each decision branch, so features that are initially nonsignificant might become so at later stages.

Network analysis (NA) also unveiled interesting information: the network of patients who had OS ≤90 days displayed significantly more connections than that of patients with OS >90 days, with distinct density of nodes in the graphs of the two populations. Of note, the appearance of significant edges between other realms and the groups “Prognostic Scores,” cachexia (“CAX24”), and quality of life (“C30”) shows how these patients can be more prone to clinical complications that may affect patient prognosis, functionality, and wellbeing.

This work also presents a plausible way to facilitate the development of ML models in the clinical healthcare setting. Robust population modeling is hindered by decentralization of data storage, incompatibility between electronic health record systems, and several barriers to the sharing of patient information.^40^, ^41^, ^42^ Synthetic data generation may help diminish this effect.

Moreover, the fact that only simple variables remained in the “lite” model makes the result of this research an accessible tool for the evaluation of borderline cases. It might help reduce the number of patients with ECOG‐PS >1 submitted to therapies with inherent toxicities, in addition to better allocating resources.

## 5 CONCLUSIONS

Machine learning and NA can be helpful tools for analyzing complex issues such as patient survival prediction. The encouraging results achieved by the ML models may be evidence that the methods used are helpful in improving current data analysis and potentially refining medical decision‐making. This is supported by biological rationale achieved both for NA and the pragmatically chosen features. Finally, the model with fewer input variables may present an accessible tool for evaluating NSCLC patients with poor PS. More extensive validation is needed before use in the clinical setting.

## AUTHOR CONTRIBUTIONS

**Mateus Trinconi Cunha:** Conceptualization (equal); data curation (equal); formal analysis (equal); investigation (equal); methodology (equal); software (equal); writing – original draft (equal); writing – review and editing (equal). **Ana Paula de Souza Borges:** Conceptualization (equal); resources (equal); writing – review and editing (equal). **Vinicius Carvalho Jardim:** Data curation (equal); software (equal); writing – review and editing (equal). **André Fujita:** Data curation (equal); software (equal); supervision (equal); writing – review and editing (equal). **Gilberto de Castro:** Conceptualization (equal); data curation (equal); formal analysis (equal); investigation (equal); methodology (equal); software (equal); writing – original draft (equal); writing – review and editing (equal).

## FUNDING INFORMATION

This work was partially funded by FAPESP (grants 2016/20187‐6, 2015/22814‐5, 2018/21934‐5, and 2019/03615‐2); and CNPq (grant 314989/2021‐8).

## CONFLICT OF INTEREST

The authors declare no conflicts of interest related to this work.

## Supporting information


Table S1.

## Data Availability

The data generated and analyzed during this study are available from the corresponding author upon reasonable request.
