# Supplementary material for: Predicting survival in metastatic non‐small cell lung cancer patients with poor ECOG‐PS: A single‐arm prospective study
Source: Cancer Med. 2022 Sep 26;12(4):5099–109. doi: 10.1002/cam4.5254 (PMC9972023; doi:10.1002/cam4.5254)
Supplement: Supplementary file 1 — Table S1. [file CAM4-12-5099-s001.docx]

Title: Predicting survival in metastatic non-small cell lung cancer patients with poor ECOG-PS: a single arm prospective study

Authors: Mateus Trinconi Cunha^1^, Ana Paula de Souza Borges^2^, Vinicius Carvalho Jardim^3^, André Fujita^3^, Gilberto de Castro Jr^1,2*^.

Affiliations:

1. Serviço de Oncologia Clínica, Instituto do Câncer do Estado de São Paulo, Hospital das Clínicas HCFMUSP, Faculdade de Medicina, Universidade de São Paulo, São Paulo, SP, BRAZIL
2. Faculdade de Medicina FMUSP, Universidade de São Paulo, São Paulo, SP, BRAZIL
3. Departamento de Ciência da Computação, Instituto de Matemática e Estatística, Universidade de São Paulo, SP, BRAZIL

*Correspondence and reprint requests to:

Prof. Dr. Gilberto de Castro Junior

Instituto do Câncer do Estado de São Paulo

Avenida Dr. Arnaldo, 251 – 5th. floor – Cerqueira César

São Paulo – SP 01246-000

Brazil

Phone +5511999685217

gilberto.castro@usp.br

ORCID 0000-0001-8765-3044

**Supplementary Data**

**Supplementary Table 1:** Table of node centrality and network structure analyses between networks of patients of >90 days OS and ≤ 90 days.

**
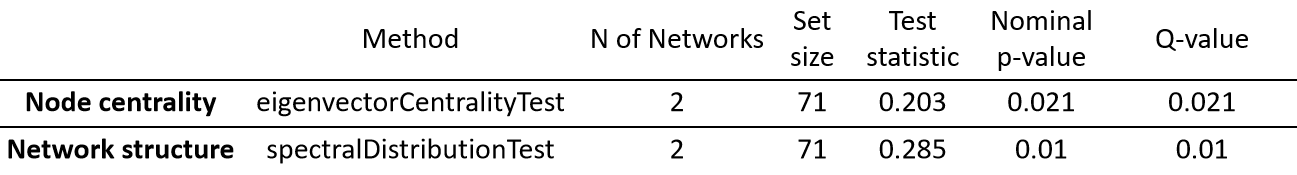
**


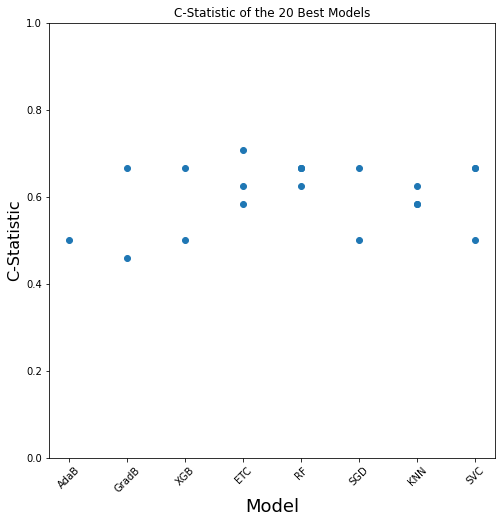


**Supplementary Figure 1:** Scatter plot of the 20 best models (classification method and hyperparameter combination) tested by the automated machine learning algorithm. Different hyperparameter configurations were used with each of the possible classification methods, resulting in more than one data point per column. AdaB = Adaptive Boosting (C-statistic: 0.500), GradB = Gradient Boosting (C-statistic: 0.458, 0.667), XGB = Extreme Gradient Boosting (C-statistic: 0.500, 0.667), ETC = ExtraTreesClassifier (C-statistic: 0.583, 0.625, 0.708), RF = Random Forest (C-statistic: 0.625, 0.667, 0.667, 0.667), SGD = Stochastic Gradient Descent (C-statistic: 0.5, 0.667), KNN = K-Nearest Neighbors (C-statistic: 0.583, 0.583, 0.625), SVC = Support Vector Classifier (C-statistic: 0.500, 0.667, 0.667).

**
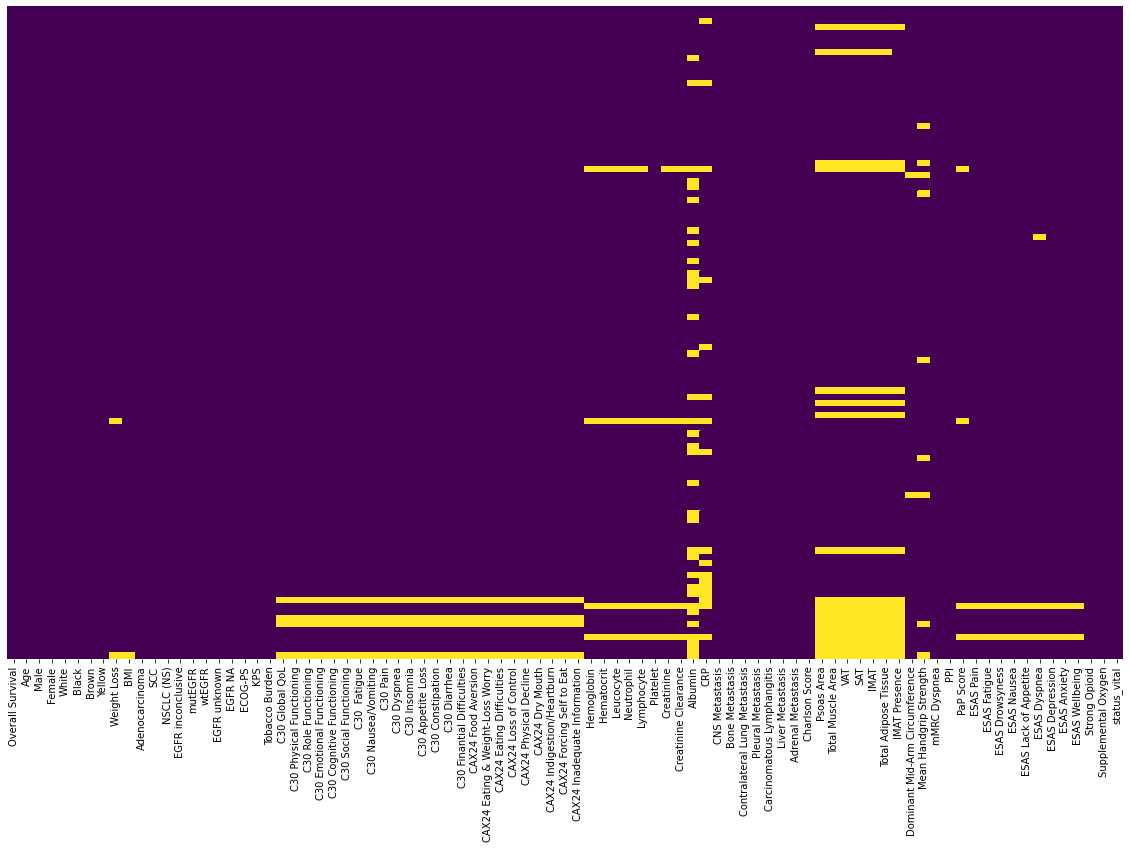
**

**Supplementary Figure 2:** Heatmap representing filled (purple) and missing (yellow) data. Y axis represents each patient, while X axis represents each variable.CRP = C-reactive protein. C30 and CAX24 features had 3.77% missing values. Laboratory exams had 3.77% missing values, except for platelet count (2.83%). Albumin had 32.08% missing values, CRP had 16.04% missing values. CT-assisted body composition measures had 16.98% missing values, mid-arm circumference, as well as ESAS evaluations had 1.89% missing values. PaP score had 3.77% missing values. ESAS depression had 2.83% missing values. Mean hand-grip strength had 8.49% missing values.

**
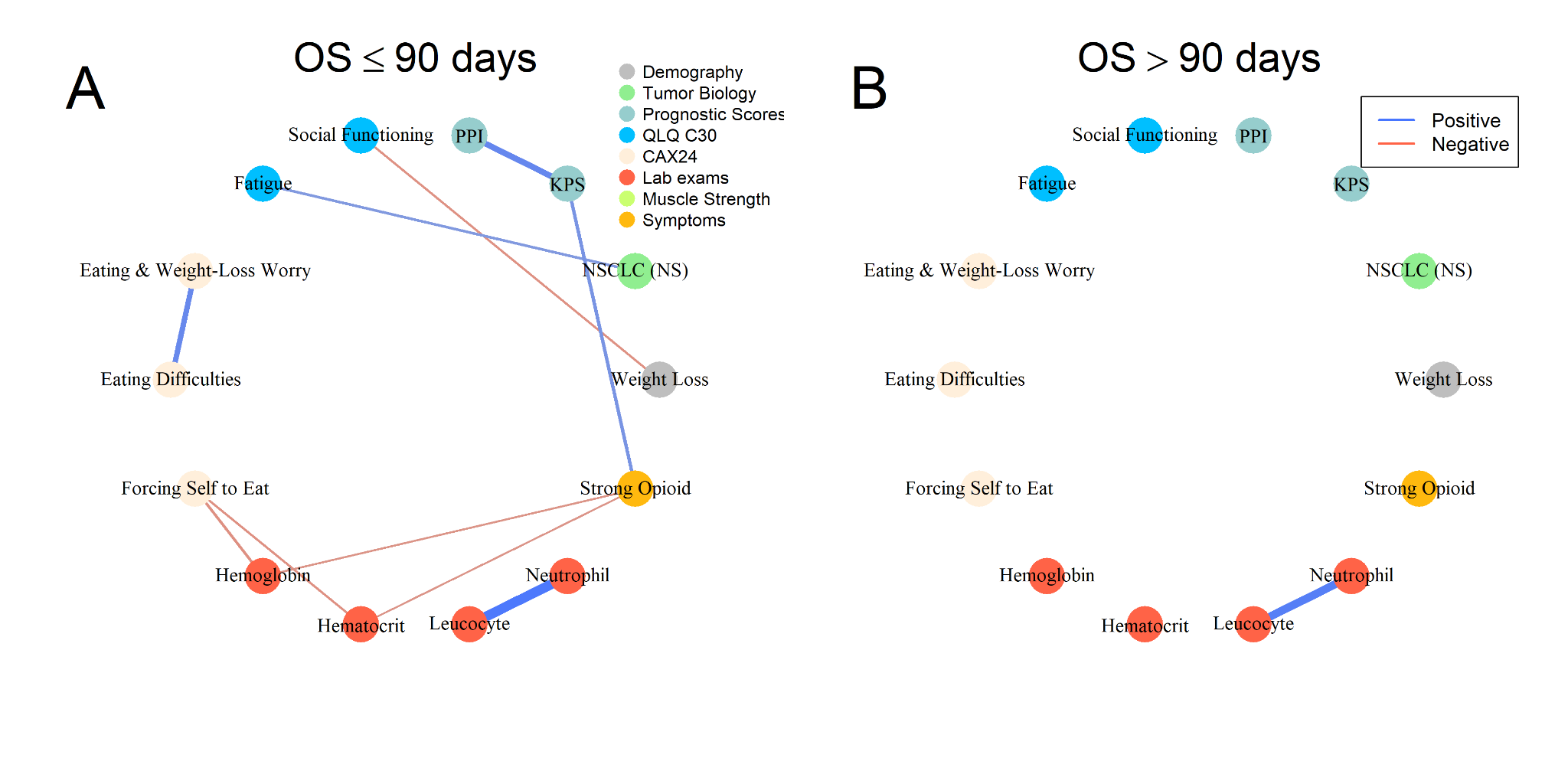
**

**Supplementary Figure 3:** Network representation of edges with statistically significant difference between patients with OS ≤ 90 days (A), and > 90 days (B). Edge color and width represent the sign and magnitude of correlations, respectively. Node color represents the features’ group and radius represents the prestige score.

**
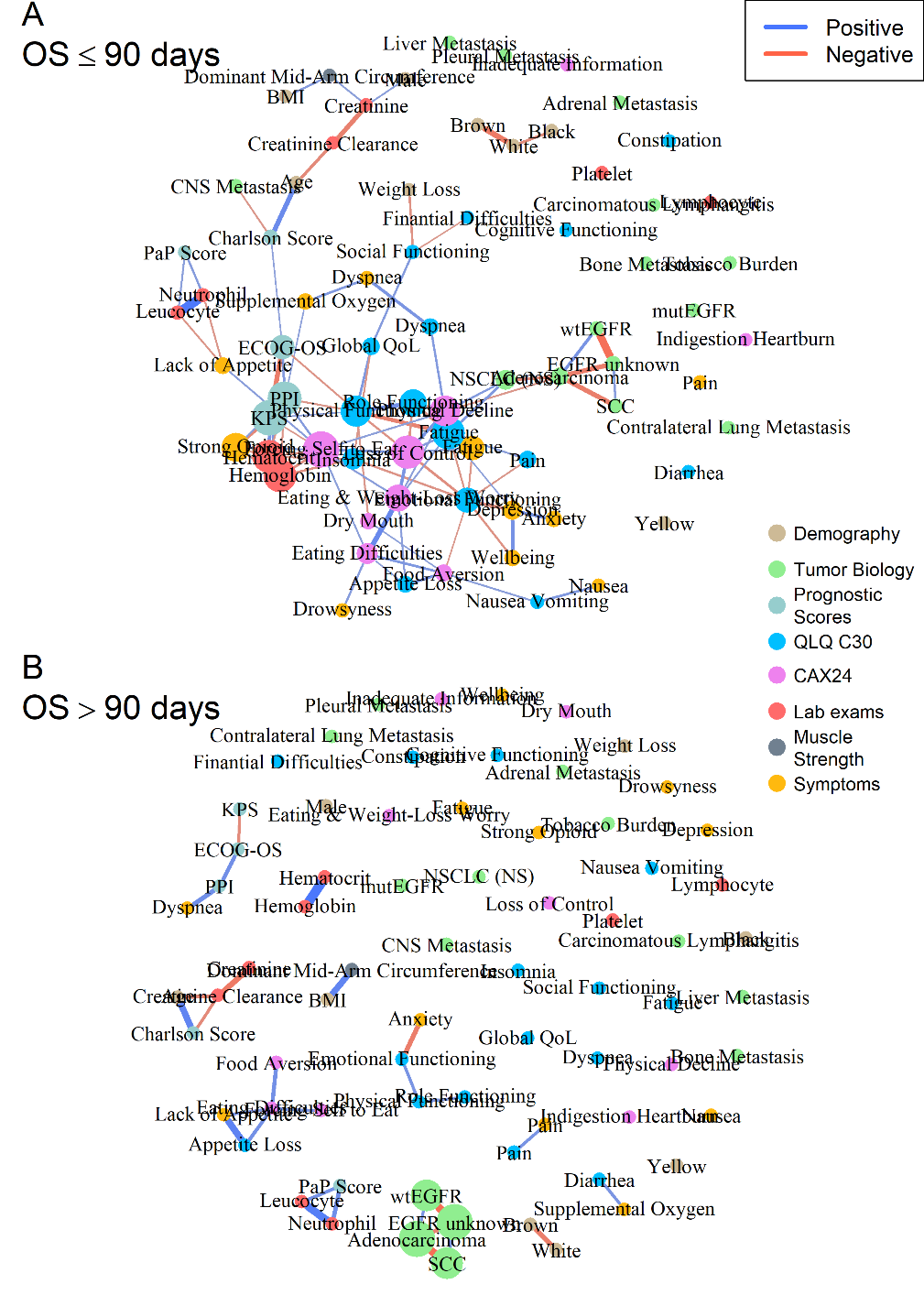
**

**Supplementary Figure 4:** Network analysis representation, with features names, for patients with OS ≤ 90 days (A), and > 90 days (B). Node colors are grouped by similarity. Edge color and width represent the sign and magnitude of correlations, respectively. Node radius represents prestige score.
